# Association of Dementia Severity and Assistances Needs on Missed Home Health Visits.

**DOI:** 10.1093/geroni/igab046.3669

**Published:** 2021-12-17

**Authors:** Sara Knox, Brian Downer, Allen Haas, Kenneth Ottenbacher

**Affiliations:** 1 Medical University of South Carolina, Charleston, South Carolina, United States; 2 University of Texas Medical Branch, Galveston, Texas, United States

## Abstract

Individuals with Alzheimer’s Disease and Related Dementias (ADRD) experience barriers to accessing health care services, including services provided during home health care. Additionally, it is not clear if people with ADRD who are admitted to home health care receive all the services needed to maximize their outcomes. Barriers to receiving the optimal care can include the presence or absence of a care giver, behavioral and psychological symptoms of ADRD, or therapists lacking the skills needed to effectively engage patients in therapy sessions. These barriers may vary dependent on the patient’s ADRD severity. The purpose of this study was to examine the relationship between dementia severity and early discharge from home health care. This was a retrospective study of 142,376 Medicare beneficiaries with ADRD who received home health care between October 2016 and September 2017. Early discharge was defined as discharge from home health with more than two missed visits. Early discharge rates were calculated, and multilevel logistic regression was used to estimate the relative risk (RR) of early discharge, by dementia severity level, adjusted for patient and clinical characteristics. 10.4% of beneficiaries had an early discharge. Dementia severity was not associated with risk of early discharge. However, level of medication assistance needed was found to be associated with risk of early discharge (RR=0.849; 95% CI 0.759 - 0.948). Medication management may impact a patient’s ability to adequately attend and engage in home health therapy services. Further studies are needed to better delineate the interaction between medication management and early discharge.

